# Testing the uniqueness of deep terrestrial life

**DOI:** 10.1038/s41598-019-51610-1

**Published:** 2019-10-23

**Authors:** Peter Trontelj, Špela Borko, Teo Delić

**Affiliations:** Department of Biology, Biotechnical Faculty, University of Ljubljana, Ljubljana, Slovenia

**Keywords:** Biogeography, Biodiversity

## Abstract

Terrestrial life typically does not occur at depths greater than a few meters. Notable exceptions are massifs of fissured rock with caves and hollow spaces reaching depths of two kilometres and more. Recent biological discoveries from extremely deep caves have been reported as sensations analogous to wondrous deep sea creatures. However, the existence of unique deep terrestrial communities is questionable when caves are understood as integral parts of a bedrock fissure network (BFN) interconnecting all parts of a massif horizontally and vertically. We tested these two opposing hypotheses – unique deep cave fauna vs. BFN – by sampling subterranean communities within the 3D matrix of a typical karst massif. There was no distinction between deep core and shallow upper zone communities. Beta diversity patterns analysed against null models of random distribution were generally congruent with the BFN hypothesis, but suggested gravity-assisted concentration of fauna in deep caves and temperature-dependent horizontal distribution. We propose that the idea of a unique deep terrestrial fauna akin to deep oceanic life is unsupported by data and unwarranted by ecological considerations. Instead, the BFN hypothesis and local ecological and structural factors sufficiently explain the distribution of subterranean terrestrial life even in the deepest karst massifs.

## Introduction

Most terrestrial life is restricted to a thin layer between the bedrock-soil interface and the canopy of the vegetation. With some tens of meters at most, or 10^−6^ to 10^−5^ of the Earth radius, the vertical dimension of terrestrial ecosystems is negligible at the global scale. It is exceeded by three orders of magnitude by the depth of marine and freshwater (including groundwater) ecosystems^1–4^. The sole exception where terrestrial environments can compete with aquatic ones are uplifted geological blocks, or massifs, of carbonate and other fissured rock with caves and hollow spaces reaching deep into their core. The deepest known caves exceed two kilometres of vertical distance from the uppermost entrance^5^. The discoveries of these and other very deep caves have opened windows into one of the least known terrestrial ecosystems on our planet^6^. Nevertheless, the increase in knowledge is slow as exploration of the deepest caves is probably no less demanding than investigation of the oceanic depths. Instructively, the deepest point of the oceanic bottom, the Challenger Deep, was reached by man in 1960, while the cave depth record is still being pushed deeper and deeper. In 2018 it was in Veryovkina Cave in the Western Caucasus, Abkhazia, Georgia, at −2,212 meters.

Discoveries of specialized invertebrates at depths of several hundred meters or more are exciting enough to be published under dazzling titles such as “the world’s deepest subterranean community^7^”, “the world’s deepest cave-dwelling centipede^8^”, or “the world’s deepest-occurring millipede^9^”. Some globally unique creatures, for example a leech with tentacles^10^ or a blind but flying insect^11^, are known exclusively from very deep caves of the Dinaric Karst in Croatia. A new amphipod family has recently been described from deep caves of the Western Caucasus^12^. This opens the obvious question whether we are witnessing the beginning of the discovery of a distinct, biologically exceptional terrestrial fauna, isolated in the inaccessible depths of karst massifs. Its marine counterpart – the remarkably adapted and scientifically challenging deep-sea fauna – is currently undergoing a new golden age of exploration^13^. Similarly, exciting discoveries have just been reported from a lake buried deep under the Antarctic ice sheet.

At about the same time as the discoveries of the deepest living terrestrial species made the news, Giachino and Vailati^14^ elaborated on a non-cave model of the subterranean environment. They had gathered extensive data on the occurrence of specialized subterranean beetles in shallow subterranean habitats formed by empty spaces between rocks right underneath the soil. As the same species were known from caves deep within karst massifs, Giachino and Vailati^14^ conjectured that the animals inhabit the fissure network that interconnects both places and that might even by their primary habitat. The idea that the entire bedrock is interwoven by cracks and fissures that form a continuous terrestrial subterranean habitat was first proposed by Racovitza^15^. As a theoretical consideration it became-well accepted (e.g.^16,17^), but received little empirical backing due to the physical inaccessibility of cave-free bedrock^18^.

**Figure 1:**
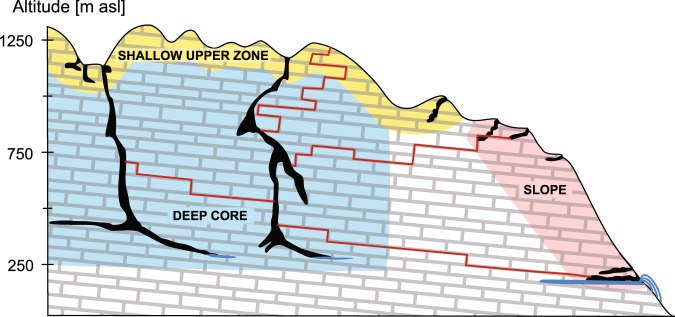
Simplified cross section of the karstic massif of Trnovski gozd with sampled caves. The explored parts of the massif are divided into three zones: the shallow upper zone (yellow), the deep core (blue), and the slope (red). The grey grid symbolizes the bedrock fissure network, which, according to several authors presents the actual habitat of subterranean fauna, while caves are just human-sized extensions. Via this network, all parts of the massif are interconnected and potentially accessible to subterranean fauna (red paths). The horizontal axis is not drawn to scale.

From the above it follows that the recent discoveries of terrestrial life at great depths can be viewed in the light of two hypotheses. The first hypothesis is that very deep caves are extraordinary geomorphological spaces that harbour an extraordinary fauna. The alternative hypothesis is that even the deepest of caves are connected to the bedrock fissure network (BFN), forming a continuous and ecologically uniform subterranean habitat all the way to the shallowest parts (Fig. 1). Subterranean species are free to migrate through the BFN and consequently no uniquely specialized deep terrestrial communities can evolve. Neither of the two hypotheses has been rigorously tested, which can be explained by the extreme technical difficulties associated with biological work in deep caves.

The basic testable implication of the BFN hypothesis is that in a continuous subterranean environment different parts should contain similar species communities. Thus, a species assemblage sampled at a point in the fissure network should be a random subsample of the overall community. Moreover, beta diversity between caves and between different zones of the massif should not be higher than expected if communities were assembled at random. Conversely, if deep caves contained a distinct fauna of their own, then exclusive, non-random species assemblages are expected along with a high beta diversity^19^.

In order to perform these tests, we explored the subterranean fauna of deep and shallow parts of a typical karst massif in its shallow upper zone, its deep core and its slopes (Fig. 1). This sampling scheme enabled us to test scenarios of vertical, horizontal, deep, shallow or general ecological connectivity within the subterranean three-dimensional matrix.

## Results

### Species distribution patterns

**Figure 2:**
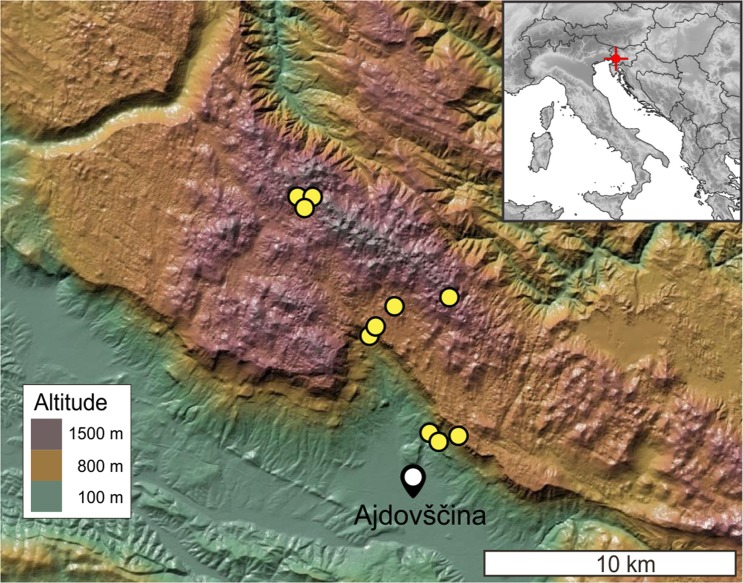
Terrain map of the study area with marked entrances of sampled caves (yellow dots) and the position of the nearby town of Ajdovščina. The position of the area in the northern Mediterranean Region is shown in the small grayscale map.

**Figure 3:**
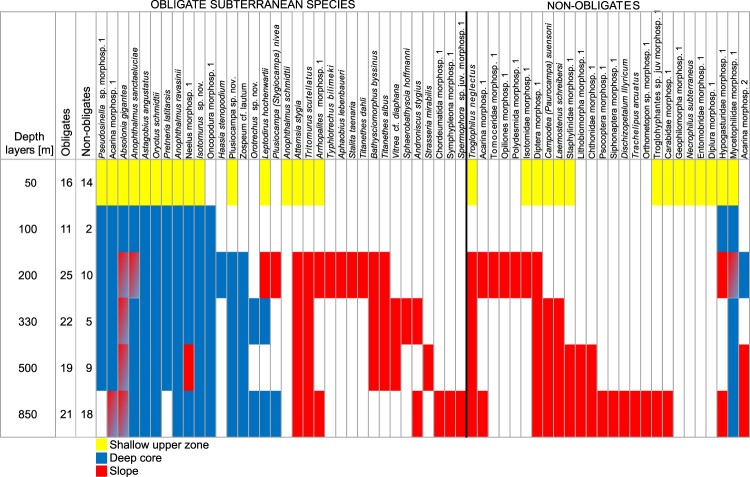
Depth profile of occurrence of obligate subterranean species in three subterranean zones of the Trnovski gozd massif. Mixed colors indicate the occurrence of a species in the same depth stratum of two different zones.

Throughout two seasons of fieldwork in the caves of the Trnovski gozd massif (Fig. 2), we collected more than 8000 individuals belonging to 60 terrestrial invertebrate species and morphospecies (Supplementary Information Tables A1 to A4). More than half (34) were obligate subterranean species. Obligate subterranean species richness was greatest (18 species) in the deep cave ‘Velika Ledena jama v Paradani’. Among the three subterranean zones of the massif, the slope zone was the most diverse (22 species), followed by 17 species in the deep core and 16 in shallow the upper zone. The shallow upper zone and the deep core had 12 (57%) obligate subterranean species in common, while only five (15%) species were shared by the deep core and the slope zone, and eight (26%) by the slope and the shallow upper zone. Among non-obligates, the percentage of shared species was highest for the shallow upper zone and the slope (39%) (Supplementary Information Fig. A1). The deep core was largely devoid of non-obligate subterranean species (Fig. 3). Every zone had one or more exclusive species.

### Similarity of subterranean communities

Analysis of similarity (ANOSIM) revealed significant differences between subterranean communities of the slope and the shallow upper zone (*R* = 0.956, *P* < 0.010) and the slope and the deep core zone (*R* = 0.936, *P* < 0.001), but not between the shallow upper zone and the deep core (*R* = 0.122, *P* = 0.180).

**Figure 4:**
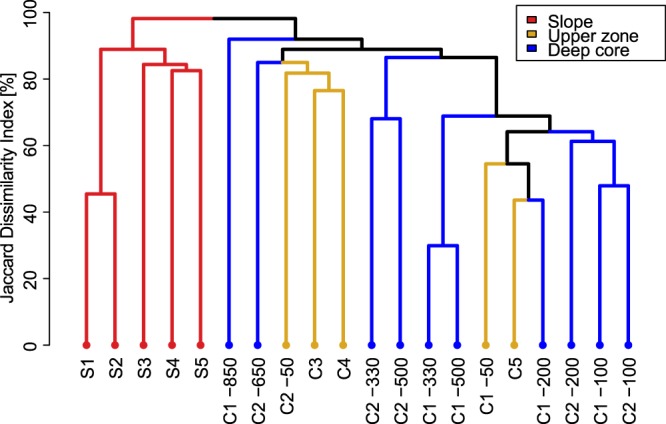
UPGMA clustering of communities of the three subterranean zones of the Trnovski gozd massif. S1–S5 caves on the slope. C1–C5 caves and sampling units in caves in the central part of massif. Cave codes as per Table 1.

The UPGMA cluster analysis showed that the similarity pattern at the level of individual sampling units was concordant with the ANOSIM results. All subterranean communities from the slope zone formed a single distinct cluster, while communities from the deep core and the upper shallow zone of the massif were intermingled in a non-predictive manner. Neither depth stratum nor geographic vicinity could consistently explain group membership (Fig. 4).

The result of the Mantel test showed a low correlation between dissimilarity of communities and spatial distance (*R* = 0.306, *P* < 0.010). We therefore concluded that any possible effect of spatial autocorrelation is negligible.

### Beta diversity partitioning

In this analysis we tested the observed overall beta diversity (BD) and its additive components nestedness and turnover against null model distributions. The null models were derived under the assumption of random movement of species and unrestricted connectivity throughout the massif. The tests were performed in a horizontal setting for each depth zone separately, and in a vertical setting for the central part and the slope of the massif.

**Figure 5:**
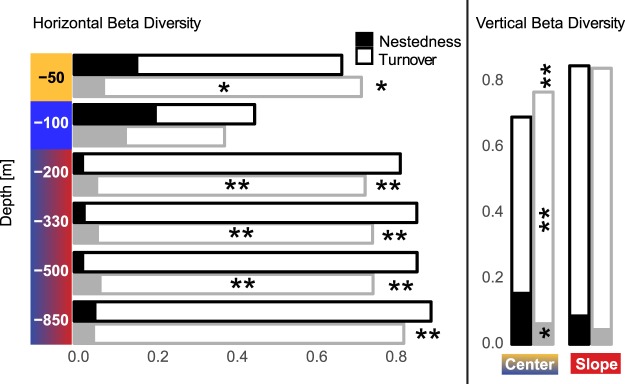
Observed (black) and modelled (light grey) beta diversity (BD) between subterranean communities partitioned into components – nestedness (full bars) and species diversity (empty bars). In the horizontal setting (left) communities are compared within a certain depth stratum, in the vertical setting (right) communities are compared within the central part and within the slope zone of the massif. Significance levels of real data vs. model comparison: **P < 0.01, *P < 0.05.

Horizontally, BD of the first two depth strata did not differ from the null model and showed a balanced composition of nestedness and turnover. The deeper strata, however, which included communities from the deep core and the slope zone, displayed a higher than expected BD with species turnover strongly predominating over nestedness (Fig. 5 and Supplementary Information Table A5).

In the vertical setting, actual BD and its components did not differ from the null model within the slope zone. In the central section of the massif that included the shallow upper zone and the deep core, vertical BD was significantly lower than predicted from the null model. This was largely the consequence of lower than expected species turnover, while the actual nestedness component was higher than in the null model.

## Discussion

Via a combination of very deep vertical caves and several smaller pits and lateral horizontal caves, we explored the interconnectedness of subterranean communities throughout the entire three-dimensional matrix of a karst massif, from its outer layers to the deep core. This new sampling approach enabled us to tackle the riddle of deep terrestrial life by comparing the observed patterns of subterranean fauna against expectations of (1) a unique deep core fauna and (2) a universal bedrock fissure network (BFN) with unrestricted connectivity. The results of our analyses offer a straightforward answer to one part of the puzzle. When looking at the depth profile of species occurrence and the similarity of communities (Figs 3 and 4; ANOSIM results), it is obvious that the communities of the deep core are not distinguishable from those of shallow caves on the top of the massif. In other words, we could find no evidence for exceptional metazoan life hidden deep inside the karst massif of Trnovski gozd.

This observation cannot be generalized for all karst massifs. Massifs that are built of less pure limestone and are consequently not so strongly karstified have less permeable fissure networks. If isolated by non-permeable rocks, they might harbour deep cave pockets with some relict species. Nonetheless, we need to highlight that the depth of caves means the vertical distance from the cave entrance and is in no way comparable to the depth of water. From the structure of porous carbonate massifs and the physical behaviour of air pressure it follows that the expected conditions in a cave section in great depth should not differ much from the conditions in a shallow cave at the same altitude. Indeed, temperatures in deeper parts of caves tend to be higher than closer to the entrance, reflecting the normal altitudinal temperature gradient^20^. Unlike the extreme pressures and low temperatures of deep seas, there are no extreme environmental cues in deep caves. These considerations alone should be enough to question the scientific justification of labels such as “the deepest living” terrestrial organism or community.

In the Trnovski gozd massif, the deep sections of the deep caves are slightly warmer than the entrance sections and caves of the shallow upper zone, where snow and ice persist year round. Still, the temperature in these caves does not exceed about 4 °C. Low temperature seems to be the most important common ecological characteristic of the upper zone of the plateau and the deep core. It is caused by the harsh climate at the high altitude of the cave entrances and the downward gravitational movement of cold air through vertical cave passages. This can explain the continuous vertical distribution of cold-adapted species such as the leiodid beetles *Astagobius angustatus* and *Pretneria latitarsis*. It is noteworthy that the terrestrial invertebrates from the extreme depths of Western Caucasian caves show the same ubiquitous vertical distribution and are not unique to the deep core zone^7,9^.

On the other hand, we found a markedly different faunal composition in caves on the south-western slopes of the massif. Their entrances are at altitudes between 250 and 900 m. Because of the lower altitude compared to the plateau-caves and because of the strong insolation of the slope these caves are much warmer. With temperatures between 7 and 10 °C they have the typical climate of temperate caves of the region. Their communities were characterized by species commonly found in the wider region, for example the carabid beetle *Typhlotrechus bilimeki*, the trichoniscid isopod *Titanethes albus* and the dysderid spider *Stalita taenaria*. Although this was not the primary question of the present study, the results suggest that differences between communities within the explored karst massif are driven by ecological influences from the surface, especially temperature, rather than by structural characteristics related to the depth of caves. This is not surprising as it has been amply demonstrated that terrestrial cave animals are often stenothermic and react to temperature differences already at the smallest of scales (e.g.^21–24^).

Our results support vertical connectivity between all parts of the massif. This is especially pronounced in the central section of the massif where species turnover between shallow and deep parts is low and the nestedness component of beta diversity is higher (Fig. 5). This means that individual cave communities from the shallow upper zone of the massif are subsamples of deep-core communities. From studies in karst hydrology and hydraulics it is known that the structure of fissure systems in the upper zones of unsaturated karstic aquifer is tree-like, causing a concentration of flow and particles in deeper zones^25^. Animals from a wider area of shallow subterranean spaces could be funnelled towards deeper parts of vertical caves by flowing water or gravity. While still compliant with the basic premises of the BFN hypothesis, this finding adds a new facet to the complexity of diversity patterns within karst massifs. It shows that deep vertical caves are more than just human-sized fissures: much to the advantage of cave biologists, they act as collectors of fauna.

Horizontal connectivity was unrestricted and thus model-compliant for all parts of the massif except between deep core and slope cave communities (Fig. 5). The deviating composition of the slope cave communities may have a purely ecological explanation (see above), but physical barriers to dispersal, for examples in the form of lithological discontinuities (e.g.^26^) or clogging of the fissures by sediments, could also play a role. To explore this possibility, we used the distribution of species as a qualitative test. Several large-bodied obligate subterranean species, most notably the leiodid beetle *Leptodirus hochenwartii*, the carabid beetle *Anophthalmus sanctaeluciae* and the onychiurid collembolan *Absolonia gigantea* were distributed in both the deep core and the slope zone. It thus seems likely that the fissure network connects the deep core with the slopes of the massif and that sufficiently mobile and ecologically tolerant species can migrate in between.

In this context we have to emphasize that very rare species are not reliable indicators of the exclusivity of communities. It is not unusual for subterranean species to be known from single individuals only or to occur with extreme irregularity^27^. Out of the four species that we found exclusively in the deep core zone (Fig. 3), three are known from several other sites in a wider area and thus cannot be considered unique to the depths of the Trnovski gozd massif. Only the undescribed carabid beetle *Orotrechus* sp.n. appears to be locally endemic. Since we were able to collect a mere three specimens despite considerable sampling efforts, it is impossible to draw conclusions about its true habitat. Nevertheless, the new *Orotrechus* species is the sole candidate for an exclusive deep cave species in our study area.

In the light of the findings presented, two recent general proposals about deep caves need to be reconsidered. The first is advocating the deep subterranean environment as a model system in global change biology on the grounds of its internal constancy and simplicity^28^. The authors propose that subterranean species can be used as *in situ* predictors of what is to be expected in a global change scenario because they cannot move to places with more suitable conditions. And, further, that “most of the environmental conditions are also virtually homogeneous through all possible microhabitats within the deepest parts of a cave system^28^”. Our own observations (see *Study area* section) and other measurements in deep cave systems^29,30^ suggest a more heterogeneous and dynamic picture. Within the massif, temperatures range from freezing to 10 °C or more, and species are free to migrate between various parts of the system and chose their optimal environment over relatively large distances. The complexity of responses of cave faunas to past and future climate change has been comprehensively reviewed by Mammola^31^.

The second proposal comes from an extensive and meticulous study of the distribution of terrestrial cave fauna in caves by Novak *et al*.^32^. They suggest that karstic massifs are inhabited not by one, but by two distinct subterranean faunas: a richer shallow one, and a less diverse deep one. The point of replacement is at about 10 meters of depth. Their interpretation is that the shallow fauna is adapted to the subsoil layer of subterranean spaces, or shallow subterranean habitat, and the “deep subterranean fauna” is adapted to live in deeper spaces. This interpretation contradicts the predictions of the BFN model that states that the shallow part of the subterranean environment forms belongs to a single spatially and ecologically continuous network of subterranean spaces. The faunal distribution in the Trnovski gozd massif does not match the pattern described by Novak *et al*.^32^, although our sampling approach was too different to allow a direct comparison. They used the term “deep cave” to denote depth greater than 10 m, while we were primarily interested in the deep core of the massif lying beneath the bottom of the entrance pits at about 100 m of depth.

## Methods

### Study area

We conducted a comprehensive survey of the terrestrial subterranean fauna of the karst massif of Trnovski gozd, Slovenia, in the northern part of the Dinaric Karst. Trnovski gozd is a typical Eastern Mediterranean karst massif, stretching some 25 km from northwest to southeast, with a plateau-like central part measuring about eight kilometres in width at its widest part. It is covered by beech-fir forest and meadows. The annual precipitation exceeds 3000 mm per year and the average annual temperatures range from 5 to 9 °C. Temperatures in the surveyed caves vary from just above 0 °C in ice caves on the top of the massif to about 4 °C at the bottom of deep vertical caves and reach up to 10 °C in low altitude horizontal caves with entrances on well-insolated slopes (own measurements).

With an altitude of 1495 m a.s.l. at the highest peak and 240 m a.s.l. at the base, the thickness of the bedrock matrix and thus the potential depth of caves exceeds one kilometre. The bedrock is predominantly formed of Mesozoic limestone and dolomite. There are approximately 200 registered caves^33^ on the massif that is known for the high diversity of subterranean beetles^34,35^.

The area constitutes a transit zone between the Mediterranean, Alpine, Dinaric and Central European biogeographic regions, and is marked by high faunal exchange (e.g.^36^).

### Sampling design

The terrestrial subterranean fauna of the Trnovski gozd massif was quantitatively assessed via 185 sampling sites distributed over 10 caves at various positions on the massif (Fig. 2), ranging from 0 to 850 m in depth, with entrances situated at altitudes from 250 to 1190 m a.s.l. (Table 1). We divided the massif into three zones: the slope zone, the shallow upper zone, and the deep core zone. Within zones, we designated sampling units that formed the basis for subsequent spatial analyses. A sampling unit constituted either an entire small cave or one of six depth strata of a deep cave. There were five sampling units in the slope zone, five in the shallow upper zone, and ten in the deep core zone. The depth strata were assigned as follows: 0–50 m, 50–100 m, 100–200 m, 200–330 m, 330–500 m, and beyond 500 m. The 0–50 m stratum was assigned to the shallow upper zone, all other strata were assigned to the deep core zone. Their boundaries roughly follow a flat exponential scale and at the same time reflect constraints set by cave physiognomy, such as deep vertical pits or narrow passages. The horizontal distance to the surface of all deep parts of the deep caves exceeds three kilometres.The layer of shallow subterranean spaces immediately under the soil was sampled at the cave entrances. To reduce sampling bias due to seasonality, the sampling was performed both in the spring/summer season of 2015 and in the winter season of 2015–2016. Samples from both seasons were pooled.

**Table 1:** Names and basic data of sampled caves of the Trnovski gozd massif.

Cave	Code	Cadastre No.*	Entrance elevation [m a.s.l.]	Total/Surveyed depth [m]	Total/Surveyed length [m]	N traps
Bela griža 1	C2	7937	1190	884/650	2054/930	44
Bošnarjev brezen	S3	782	895	56/56	130/100	12
Gorjanka	S5	11139	855	27/27	80/30	6
Veliki Hubelj	S2	2880	260	40/0	440/20	6
Jama pri Mali Ledenici v Paradani	C4	922	1140	25/25	235/40	6
Ledenica pri Dolu	C5	751	995	80/30	180/130	12
Mala ledena jama v Paradani	C3	750	1150	69/20	69/30	8
Pajkova reža	S1	6122	250	16/16	330/60	10
Tunel	S4	8862	720	13/13	40/30	6
Velika ledena jama v Paradani	C1	742	1135	858/850	7311/1700	75

*Karst Research Institute (2018); S1–S5: caves on slope; C1–C5: caves of central part of massif.

### Trapping and sample processing

We used baited pitfall traps with a supersaturated sodium chloride solution as fixative. Baits were made of a mixture of decaying poultry and cheese. Traps consisted of 0.2 litre one-way PVC drinking cups, filled with fixative to ¼, and a tube containing the bait. Each trap was inserted into a natural or dug hole so that it did not protrude from the surrounding ground. Altogether, 185 traps were set and examined. Traps were placed along the cave passages as evenly as possible, from the entrances to the most remote parts, and left for approximately 30 days. The trapped animals were determined to the level of species or morphospecies where an exact taxonomic assignment was not possible (e.g. because of the lack of experts or determination keys). Specimens that were too poorly preserved for taxonomic identification were excluded from all further analyses. All species were assigned to the two major ecological groups commonly used to assess subterranean biodiversity (e.g.^32,37^): obligate subterranean species or troglobionts, and non-obligates or non-troglobionts (Supplementary Information Tables A1 and A2). For this assignment we used mainly literature data, database entries^38^, and traits associated with subterranean life, such as loss of eyes and body pigment, or, when compared to close surface relatives, elongated appendages.

In recent years, researchers have undertaken considerable efforts to improve and standardize sampling techniques for terrestrial cave fauna^39–41^. In addition to recommendations from these studies, we had to consider special circumstances resulting from the depth, physiognomy and objective dangers of alpine vertical caves, where large-scale systematic biological surveys have not yet been performed. These factors included the danger of falling rocks, avalanches and flooding. Technical limitations prevented sampling in vertical sections with free-hanging rope. Finally, exposure and exhaustion of the researchers precluded long and meticulous searching-by-eye and dictated a trapping-oriented sampling approach.

### Normalization of abundance data

For reasons mentioned above, the number of traps set per meter of cave could not be held constant. To account for the unevenness in sampling density we applied a twofold normalization procedure by which we obtained comparable quantitative data for each sampling unit (see *Sampling design*). We merged all samples collected in a single sampling unit and corrected total abundances for the number of traps per meter of cave passage length. This normalized abundance matrix [individuals of a species per sampled trap per meter] was then used for further quantitative analyses. All quantitative analyses were performed on obligate subterranean species only.

### Analyses of communities

Species assemblages of obligate subterranean species, or subterranean communities, were compared based on the incidence-based Jaccard dissimilarity index^42^. In order to test the notion of a single continuous subterranean habitat throughout the massif, we compared species compositions of communities from the shallow upper zone, the deep core zone, and the slope zone. We used pairwise Analysis of similarities (ANOSIM) to test whether the similarity between communities within zones was equal to the similarity of communities between different zones. We applied the Jaccard distance matrix to derive distance ranks that were in turn used to calculate the test statistic *R*.

Next, we performed a hierarchical cluster analysis of subterranean communities from individual sampling units in order to explore whether there were any similarity patterns at finer scales. We applied the unweighted pair group method with arithmetic average (UPGMA) using the Jaccard index as measure of pairwise similarity.

We explored the risk of spatial autocorrelation influencing the analyses of dissimilarity patterns using the Mantel test with Spearman correlation, which is appropriate for non-linear data. By running 999 permutations of our original pairwise matrices of geographic distances and dissimilarities of communities, we tested whether these two measures were more strongly correlated than expected by chance. Geographical distances were calculated as Euclidean distance between caves or sampling units in the three-dimensional space of the massif.

### Beta diversity analysis

Beta diversity (BD) was used to measure the contribution of the diversity among sampling units to the total species diversity of the massif or selected zone of the massif. Differences between communities can emerge from two processes: species turnover (species replacement between one site and another) and nestedness (species losses at one of the sites). Total BD is the sum of both components^43,44^. Particularly instructive tests of ecological and distributional hypotheses can be derived from comparing observed BD values and their components to the values expected under a null-model of random migration of species between sampling units^45^.

Our tests of BD components rested on the assumption that turnover should predominate if deep cave communities were composed of unique species and were distinct from communities in the shallow upper zone and the slope zone. Also, turnover should be the main BD component between sampling units if these were somehow isolated from each other or if they differed ecologically and therefore supported different communities. Conversely, nestedness should prevail in cases where dispersal was possible but mostly unidirectional, as for example from shallower parts of the massif to deeper ones. To test these assumptions, we analysed vertical and horizontal BD patterns within the massif. Vertical BD was calculated as average diversity among all possible combinations of communities from the shallow upper zone and the pooled communities of the deep core, resulting in overall 10 combinations of one shallow unit and five deeper sampling units each. For slope communities we had only one assemblage per elevation and consequently could calculate only one multiple-site BD value. Horizontal BD was calculated separately for each depth stratum of the massif (see stratification scheme on Fig. 3) as the diversity among communities lying in the same stratum, regardless of their lateral position in the massif.

We generated 9,999 null models for each combination of communities. Following the approach of Wright *et al*.^46^, the overall diversity of the massif was held constant and the number of occurrences per sampling unit was not limited. The observed BD patterns were tested against BD calculated from null model distributions.

For BD analysis we used only presence-absence data. Interpretation and null model testing of abundance-based BD patterns are a matter of ongoing debate and yet to be fully developed and tested^47,48^. We opted for a proven, qualitative approach also because of the extreme nature of subterranean habitats, where animals are often rare and hard to sample with constant effort over space^41^.

All analyses were carried out within R framework^49^ using the VEGAN package^42^.

## Supplementary information

Supplementary material

## Data Availability

The data and code used to conduct this research are available via the Zenodo open-access repository at 10.5281/zenodo.2677708.
