# Supplementary material for: Testing the uniqueness of deep terrestrial life
Source: Sci Rep. 2019 Oct 23;9:15188. doi: 10.1038/s41598-019-51610-1 (PMC6811635; doi:10.1038/s41598-019-51610-1)
Supplement: Supplementary file 1 — Supplementary material [file 41598_2019_51610_MOESM1_ESM.pdf]

Testing the uniqueness of deep terrestrial life

Peter Trontelj, Špela Borko and Teo Delić

Table A1: Number of specimens of obligate subterranean species per cave

Species	Bela Griža 1	Velika ledena jama v Paradani	Bošnarjev brezen	Ledenica pri Dolu	Mala ledena jama v Paradani	Jama pri Mali Ledenici v	Tunel	Gorjanka	Pajkova Reža	Veliki Hubelj
Absolonia gigantea	17	675	46	35	2		15	63		10
Acarina morphosp. 1	29	82		6	21	5			1	
Androniscus stygius								21		2
Anophthalmus ravasinii	1	42		30		1				
Anophthalmus sanctaeluciae	154	32	1	4						
Anophthalmus schmidtii						1				
Aphaobius lebenbaueri			33							
Arrhopalites sp. morphosp. 1			1			52			5	
Astagobius angustatus	268	682		174	56	20				
Attemsia stygia		1	12		1	8	9	19	2	
Bathysciomorphus byssinus			40				234	25		
Chordeumatida morphosp. 1										1
Haasia stenopodium		1								
Isotomurus sp. nov.	85	121		7						
Leptodirus hochenwartii	1	1	18	1		1				
Neelus sp. morphosp. 1	2	2				1	4			
Oncopodura morphosp. 1	244	7								
Orotrechus sp. nov.	2									
Oryotus schmidtii	712	155		41	15	55				
Plusiocampa (Stygiocampa) nivea	18	125	219							
Plusiocampa sp. nov.	17	75		11						
Pretneria latitarsis	3	33		64	33	13				
Pseudosinella sp. morphosp. 1	436	1		11						
Spermophora sp. juv. morphosp. 1									1	
Sphaerobathyscia hoffmanni								29		
Stalita taenaria			1							
Strasseria mirabilis							1			
Symphypleona morphosp. 1									1	
Titanethes albus			23				2	15		
Titanethes dahli			1							
Tritomurus scutellatus	1	1	20	2		14	62	215	36	88
Typhlotrechus bilimeki			5							
Vitrea cf. *diaphana*							15	2		
Zospeum cf. *lautum*	3	2								
**SUM**	**1993**	**2038**	**420**	**386**	**128**	**171**	**342**	**389**	**46**	**101**

Table A2: Number of specimens of non-obligate subterranean species per cave

Species	Bela Griža 1	Velika ledena jama v Paradani	Bošnarjev brezen	Ledenica pri Dolu	Mala ledena jama v Paradani	Jama pri Mali Ledenici v	Tunel	Gorjanka	Pajkova Reža	Veliki Hubelj
Acarina morphosp. 1		2					6		6	
Acarina morphosp. 2			7						4	
Campodea (Paurocampa) suensoni						10	11	10	130	212
Carabidae morphosp. 1	3	7			8	6			10	
Chthonidae morphosp. 1							2			4
Diplura morphosp. 1		1								
Diptera morphosp. 1	2		102		7	7	30	90	121	45
Dischizopetalum Illyricum									3	
Entomobridae morphosp. 1	60									
Geophilomorpha morphosp. 1						1				
Hypogasturidae morphosp. 1	2		2						7	
Isotomidae morphosp. 1	47		17		220					
Laemostenus schreibersi		5				26	19	76	168	8
Lithobiomorpha morphosp. 1							2		5	
Mycetophilidae morphosp. 1	8	199	18	83	1					
Necrophilus subterraneus					4	5				
Opiliones morphosp. 1			1							
Orthometopon sp. morphosp. 1									29	
Polydesmida morphosp. 1			2							
Psocoptera morphosp. 1									19	
Siphonaptera morphosp. 1									1	
Staphylinidae morphosp. 1	13				1		3		32	7
Tomoceridae morphosp. 1			8							
Trachelipus arcuatus									8	
Troglohyphantes sp. juv morphosp. 1						1			3	1
Troglophilus neglectus			1			6	12	43	114	
**SUM**	**135**	**214**	**158**	**83**	**241**	**62**	**85**	**219**	**660**	**277**

Table A3: Number of specimens of all species per depth layers – Velika ledena jama v Paradani

	0 - 50 m	50 - 100 m	100 - 200 m	200 – 330 m	330 – 500 m	500 – 850 m
**non-obligate subterranean species**						
Acarina morphosp. 2			2			
Carabidae morphosp. 1	7					
Diplura morphosp. 1	1					
Laemostenus schreibersi	5					
Mycetophilidae morphosp. 1	11	1	28	11	24	124
Onychiuridae morphosp. 1	1					
**obligate subterranean species**						
Absolonia gigantea	1	1	27	39	89	518
Acarina morphosp. 1	10	11	10	5	22	24
Anophthalmus ravasinii		4	14	10	9	5
Anophthalmus sanctaeluciae	4	3	1	2	2	20
Astagobius angustatus	273	111	152	34	63	49
Attemsia stygia	1					
Haasia stenopodium			1			
Isotomurus sp. nov.			29	36	35	21
Leptodirus hochenwartii						1
Neelus sp. morphosp. 1				1		1
Oncopodura morphosp. 1						7
Oryotus schmidtii	99	26	12	3	9	6
Plusiocampa (Stygiocampa) nivea						125
Plusiocampa sp. nov.			1	2	2	70
Pretneria latitarsis	21	9	2		1	
Pseudosinella sp. morphosp. 1	1					
Tritomurus scutellatus	1					
Zospeum cf. lautum					1	1

Table A4: Number of specimens of all species per depth layers – Bela Griža 1

	0 - 50 m	50 - 100 m	100 - 200 m	200 – 330 m	330 – 500 m	500 – 650 m
**non-obligate subterranean species**						
Carabidae morphosp. 1	3					
Diptera morphosp. 1	2					
Entomobridae morphosp. 1	60					
Hypogasturidae morphosp. 1	1	1				
Isotomidae morphosp. 1	47					
Mycetophilidae morphosp. 1	3	3		1		1
Staphylinidae morphosp. 1	13					
**obligate subterranean species**						
Absolonia gigantea		2	15			
Acarina morphosp. 1	18	6	3	1	1	
Anophthalmus ravasinii		1				
Anophthalmus sanctaeluciae	3	3	5	129	13	1
Astagobius angustatus	3	41	28	146	47	3
Isotomurus sp. nov.		3	9	53	20	
Leptodirus hochenwartii				1		
Neelus sp. morphosp. 1		1	1			
Oncopodura morphosp. 1		3	3	100	114	24
Onychiuridae morphosp. 1	89	286	15			
Orotrechus sp. nov.				1		1
Oryotus schmidtii	15	14	16	518	126	23
Plusiocampa (Stygiocampa) nivea						18
Plusiocampa sp. nov.	6		4	7		
Pretneria latitarsis	1		1	1		
Pseudosinella sp. morphosp. 1	14	5	9	357	51	
Tritomurus scutellatus	1					
Zospeum cf. lautum			1	2		

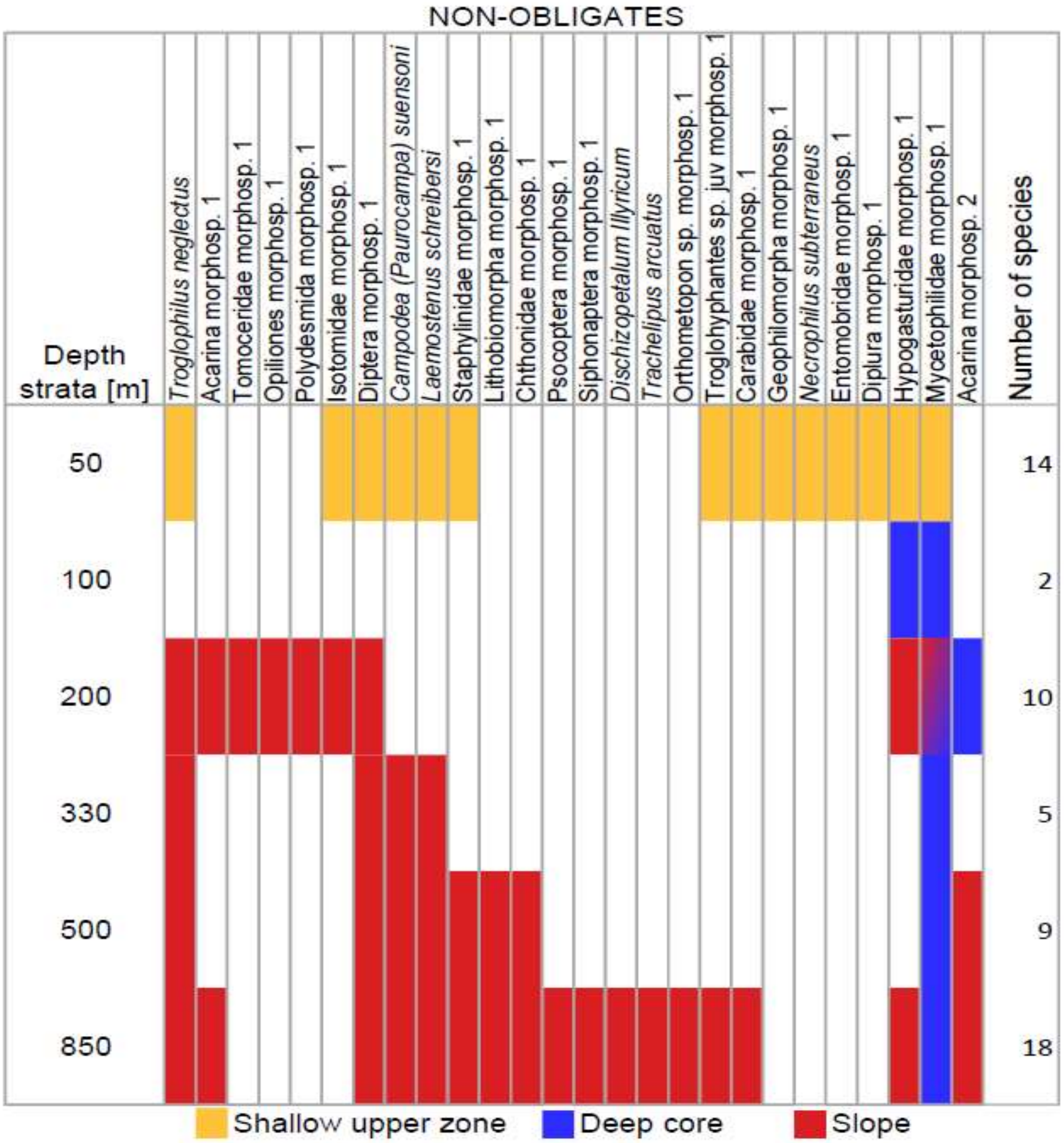

Figure A1: Altitudinal profile of species occurrence in three subterranean zones of the Trnovski gozd massif, non obligate subterranean species.

Table A5: Results of beta diversity analysis. Overall beta diversity is coded as β_{JAC}, nestedness as β_N and turnover as β_T. *p*-values lower than 0.05 are bolded.

HORIZONTAL BETA DIVERSITY

	Real Data			Null Model			p-VALUES		
Depth stratum	β_T	β_N	β_{JAC}	β_T	β_N	β_{JAC}	β_T	β_N	β_{JAC}
-50	0.516	0.158	0.674	0.649	0.074	0.723	**0.015**	0.050	**0.038**
-100	0.250	0.205	0.455	0.250	0.129	0.379	1.000	0.624	1.000
-200	0.800	0.021	0.821	0.676	0.057	0.733	**0.010**	0.326	**0.007**
-330	0.837	0.026	0.863	0.693	0.059	0.752	**0.003**	0.411	**0.003**
-500	0.842	0.022	0.864	0.687	0.065	0.753	**0.003**	0.330	**0.005**
-650	0.846	0.053	0.899	0.781	0.049	0.830	0.084	0.738	**0.002**

VERTICAL BETA DIVERSITY – CENTER

	Real Data			Null Model			p-VALUES		
Combination	β_T	β_N	β_{JAC}	β_T	β_N	β_{JAC}	β_T	β_N	β_{JAC}
A	0.389	0.215	0.604	0.667	0.070	0.737	**0.000**	**0.001**	**0.000**
B	0.535	0.132	0.667	0.710	0.061	0.771	**0.001**	**0.028**	**0.000**
C	0.500	0.158	0.658	0.694	0.065	0.759	**0.001**	**0.014**	**0.000**
D	0.435	0.223	0.658	0.682	0.070	0.752	**0.000**	**0.001**	**0.000**
E	0.586	0.110	0.696	0.719	0.058	0.776	**0.001**	0.064	**0.000**
F	0.543	0.154	0.698	0.681	0.067	0.749	**0.003**	**0.019**	**0.005**
G	0.548	0.162	0.710	0.706	0.064	0.770	**0.001**	**0.009**	**0.001**
H	0.581	0.156	0.737	0.719	0.061	0.780	**0.002**	**0.008**	**0.009**
I	0.578	0.143	0.721	0.722	0.059	0.780	**0.001**	**0.012**	**0.001**
J	0.667	0.096	0.763	0.745	0.053	0.797	**0.020**	0.085	**0.015**
Average	0.536	0.155	0.691	0.704	0.063	0.767	**0.003**	**0.024**	**0.003**

VERTICAL BETA DIVERSITY - SLOPE

	Real Data			Null Model			p-VALUES		
Combination	β_T	β_N	β_{JAC}	β_T	β_N	β_{JAC}	β_T	β_N	β_{JAC}
SLOPE	0.761	0.086	0.847	0.795	0.045	0.839	0.260	0.101	0.709
